# Successful Aging Among Older Hispanics

**DOI:** 10.1093/geroni/igaa057.3370

**Published:** 2020-12-16

**Authors:** Marlen Garcia, Alejandra Morlett Paredes, Dilip Jeste, Alison Moore, Maria J Marquine

**Affiliations:** 1 San Diego State University, San Ysidro, California, United States; 2 University of California, San Diego, La Jolla, California, United States

## Abstract

Successful Aging has been defined as the absence of objective physical, cognitive, and social difficulties. More recently, self-rated successful aging (SRSA) has been recognized as an important outcome in its own right. The purpose of this study was to assess SRSA and its correlates among older Hispanics/Latinos. Seventy-four Hispanic/Latino adults age 50+ (31.9% primarily Spanish-speaking; 62.5% women, mean age=69.6±12.2, mean years of education=14.3±3.3) completed a measure of SRSA (scaled from 1 [lowest] to 10 [highest]), and self-report measures of hypothesized correlates, including culturally-relevant factors (language use, acculturation, fatalism, familism, perceived discrimination and frame of reference), as well as physical (perception of physical health and physical performance), cognitive (perception of cognitive problems), and psychosocial correlates (social functioning and resilience). Fifty-five percent of the participants reported SRSA of 8 or above (mean=7.99±, range: 3-10). Factors that were significantly associated with SRSA in univariable models, were entered into a multiple linear regression on SRSA. The final multivariable model explained 58.5% of the variance on SRSA (F(3,54)=27.8, p<.001) and showed that social functioning (B=.21; p=.031), resilience (B=.34; p=.002), and perception of physical health (scaled from 1 [highest] to 5 [lowest]), (B=-.43; p<.001) were independent predictors of SRSA. Culturally-relevant factors were not independently associated with SRSA in the multivariable model. While future longitudinal studies would be better suited to address causality, the present cross-sectional findings indicate psychosocial correlates of SRSA are as important as physical correlates among older Latinos. Future studies might examine whether culturally relevant factors modify these associations.

